# Bispecific antibodies targeting BCMA or GPRC5D are highly effective in relapsed myeloma after CAR T-cell therapy

**DOI:** 10.1038/s41408-024-01197-2

**Published:** 2024-12-05

**Authors:** Maximilian Merz, Danai Dima, Hamza Hashmi, Nausheen Ahmed, Friedrich Stölzel, Tobias A. W. Holderried, Roland Fenk, Fabian Müller, Natalia Tovar, Aina Oliver-Cáldes, Kristin Rathje, James A. Davis, David Fandrei, Vladan Vucinic, Soraya Kharboutli, Ben-Niklas Baermann, Francis Ayuk, Uwe Platzbecker, Anca-Maria Albici, Nathalie Schub, Friederike Schmitz, Leyla Shune, Jack Khouri, Faiz Anwer, Shahzad Raza, Joseph McGuirk, Zahra Mahmoudjafari, Kimberly Green, Cyrus Khandanpour, Marcel Teichert, Barbara Jeker, Michele Hoffmann, Nicolaus Kröger, Bastian von Tresckow, Carlos Fernández de Larrea, Thomas Pabst, Al-Ola Abdallah, Nico Gagelmann

**Affiliations:** 1 Department of Hematology, Cellular Therapy, Hemasteseology and Infectious Disease, University Hospital of Leipzig and Fraunhofer IZI, Leipzig, Germany; 2 Cleveland Clinic, Cleveland, OH USA; 3 US Myeloma Innovations Research Collaborative (USMIRC), Kansas City, MO USA; 4 The Medical University of South Carolina, Charleston, SC USA; 5 Memorial Sloan Kettering Cancer Center, New York City, NY USA; 6 Division of Hematologic Malignancies & Cellular Therapeutics, University of Kansas Cancer Center, Westwood, KS USA; 7 University Hospital Schleswig-Holstein Kiel, Kiel University, Kiel, Germany; 8 Department of Hematology, Oncology, Stem Cell Transplantation, Immune and Cell Therapy, Clinical Immunology and Rheumatology, University Hospital Bonn, Bonn, Germany; 9 Heinrich Heine University, University Hospital Düsseldorf, Düsseldorf, Germany; 10 Department of Internal Medicine 5, Haematology and Oncology, University Hospital of Erlangen, Friedrich-Alexander University of Erlangen-Nuremberg (FAU), Erlangen, Germany; 11 Hospital Clínic de Barcelona. IDIBAPS. University of Barcelona, Barcelona, Spain; 12 University Medical Center Hamburg-Eppendorf, Hamburg, Germany; 13 Campus Lübeck, University Cancer Center Schleswig-Holstein and University of Lübeck, Lübeck, Germany; 14 Department of Medicine II, Division for Stem Cell Transplantation and Cellular Immunotherapy, Universitätsklinikum Essen, Essen, Germany; 15 Department of Medical Oncology, University Hospital Inselspital and University of Bern, Bern, Switzerland

**Keywords:** Translational research, Myeloma, Immunotherapy

## Abstract

Despite the astonishing outcomes after chimeric antigen receptor (CAR) T-cell therapy for relapsed refractory multiple myeloma (RRMM), most patients eventually relapse. There are only limited data available on salvage therapies following relapse after BCMA-directed CAR T-cell therapy. Here, we analyzed outcomes of post-CAR T-cell therapy relapse and impact of different salvage strategies in an international cohort of 139 patients (*n* = 130 ide-cel, *n* = 9 cilta-cel), receiving talquetamab (*n* = 28), teclistamab (*n* = 37), combinations of immunomodulating drugs (IMiDs), proteasome inhibitors (PIs) or CD38 monoclonal antibodies (*n* = 43), and others (*n* = 31). The median time to relapse after CAR T-cell therapy was 5 months, 53% had the extramedullary disease (EMD) at relapse, associated with dismal post-relapse outcome (*P* = 0.005). Overall response and complete response upon salvage therapies were 79% and 39% for talquetamab, 64% and 32% for teclistamab, 30% and 0% for IMiDs/PIs/CD38, and 26% and 3% for others (*P* < 0.001). Duration of response, as well as median survival, was significantly improved with bispecific antibodies (*P* < 0.001, respectively). Bispecific antibodies seemed to overcome the poor prognosis associated with early relapse and EMD, and were independent predictors for improved survival in multivariable analysis. In summary, these results suggest bispecific antibodies as the standard of care for relapse after CAR T-cell therapy for RRMM.

## Introduction

Chimeric antigen receptor (CAR) T-cells have revolutionized the treatment of triple-class exposed (TCE) patients with relapsed/refractory multiple myeloma (RRMM) [1, 2]. Idecabtagene vicleucel (ide-cel) and ciltacabtagene autoleucel (cilta-cel) are currently approved for the treatment of patients with TCE RRMM after three or four lines of therapy in the European Union and the United States, respectively [3, 4]. Recent analyses demonstrated that the application of ide-cel and cilta-cel outside clinical trials achieves results that are comparable to the findings of the pivotal KarMMa and CARTITUDE-1 studies that led to the approval of the respective agents [5, 6]. Despite the unprecedented rates of deep remissions in TCE RRMM, virtually all patients experience relapse after CAR T-cell infusion and there is a substantial number of patients with primary refractory disease [7]. Several studies characterized risk factors and deciphered modes of resistance associated with dismal outcome after CAR T-cell therapies directed against BCMA. Biallelic loss of BCMA, (CAR) T-cell exhaustion and an immunosuppressive microenvironment have been identified as possible mechanisms that lead to primary refractoriness or relapse [8]. Importantly, some of these mechanisms have implications for subsequent salvage therapies, like the irreversible loss of BCMA that also causes refractoriness to BCMA-directed bispecific antibodies. Nevertheless, there are only limited data available on salvage therapies following relapse after BCMA-directed CAR T-cells. Factors associated with prolonged post-relapse survival are currently unknown [9]. In the current study, we analyzed a large, international cohort of patients treated for relapse after BCMA-directed CAR T-cell therapies for TCE RRMM. We aimed at identifying the most promising salvage options and characterizing factors associated with prolonged post-relapse survival.

## Methods

In this multicenter retrospective observational study, we included only patients infused with commercially available BCMA-directed CAR T cells for RRMM across international centers (7 from Germany, 1 from Spain, 1 from Switzerland, and 3 from the United States). Lymphodepletion with fludarabine and cyclophosphamide was administered in accordance with manufacturers’ recommendations. Out-of-specification use was allowed to reflect real-world applications. Detailed information on salvage regimens after CAR T cell relapse was obtained, with date of salvage administration, regimen used, and response to salvage. Detailed information was obtained until at least third line of salvage treatment. Administration and schedule of each salvage regimen was as per center’s discretion.

### Ethics approval and consent to participate

Each center obtained informed consent per institutional requirements and the study was approved by local ethics committees of participating centers. This study is in accordance with the Declaration of Helsinki.

### Clinical assessment and definitions

Cytokine release syndrome (CRS) and immune effector cell associated neurologic syndrome (ICANS) were assessed in accordance with official criteria [10]. Treatment response was assessed in accordance with the International Myeloma Working Group Criteria [11], but assessment and fulfillment of all response criteria remained investigator’s discretion, as was the assessment and determination of measurable residual disease (MRD). Responses were classified as: complete response (CR), very good partial response (VGPR), partial response (PR), and less than PR. Extramedullary disease (EMD) was defined as organ manifestation, assessed with CT scan, MRI or PET/CT as per each center’s policy, and sole para-osseous involvement was excluded from that definition, as it was shown consistently to be associated with similar outcomes than only marrow-involved myeloma and overall better outcomes than actual EMD [6, 12–14].

### Statistical analysis

First, we described characteristics and outcomes of patients with relapse or progression after first CAR T-cell infusion. Second, we described characteristics and outcomes of different treatment strategies as salvage after relapse post-CAR T. Third, we aimed to identify subgroups benefitting the most from certain salvage approaches, as well as predictors of outcome.

The distribution of patient and treatment characteristics was examined in the entire cohort and compared between treatment strategies and relapse type, using Chi-squared for categorical variables and Mann-U-Whitney test for continuous variables. Relapse/progression was defined as previously described [11]. Kaplan-Meier estimates were used for analysis of progression-free survival (PFS) and overall survival (OS). Duration of response (DOR) was defined as the time from response to salvage therapy until progression or relapse, death, or last follow-up. Time-to-event calculations were started from first salvage administration. Regression modelling with respect to relapse was applied within a competing risks framework by using the Fine & Gray method, with death without relapse/progression as a competing event. All analyses were conducted using R (Version 4.0.5).

## Results

### Characteristics and outcomes of post-CAR T relapse

In this large multicenter international study, we included a total cohort of 139 patients relapsed after CAR T. Most patients (94%) had received ide-cel, while nine patients (6%) had received cilta-cel.

The median follow-up from first salvage for the entire cohort was 8.6 months (95% CI, 7.6–9.6 months), and the median OS was 9 months (95% CI, 4–14 months; Appendix Fig. 1A). The median time from CAR T-cell infusion to relapse/progression was 5 months (range, 0.4–33 months), and earlier relapse within 5 months after CAR T-cell infusion was significantly associated with worse OS (*P* = 0.002), with a median OS of 4.9 months (95% CI, 3.5–6.3 months) versus median OS not reached for patients with late relapse more than 5 months after CAR T-cell infusion (Appendix Fig. 1C).

Half of the total cohort (53%) showed relapse with EMD, and extramedullary relapse was significantly associated with dismal post-relapse outcome (*P* = 0.005), with a median OS of 5 months (95% CI, 3–7 months) versus median OS not reached for patients with non-EMD relapse (Appendix Fig. 1B).

The MyCARe model, initially developed from time of CAR T-cell infusion for relapse/progression and PFS prognostication [6], also differentiated 3 groups with distinct outcome after first salvage, with median OS not reached for the low risk group versus 10 months (95% CI, 2–17 months) for the intermediate risk group versus 4 months (95% CI, 3–5 months) for the high risk group (*P* = 0.02; Appendix Fig. 1D).

### Characteristics of salvage strategies

Patients received the following salvage treatments for relapse/progression: talquetamab (20%), teclistamab (27%), combinations of immunomodulatory drugs (IMiDs), proteasome inhibitors (PIs) or CD38 monoclonal antibodies (31%), chemotherapy (11%), radiotherapy (3%), autologous or allogeneic transplant (3%), and others. For better comparability, we categorized the regimens into 4 groups: talquetamab, teclistamab, combinations of IMiDs/PIs or CD38 monoclonal antibodies, and others. Patient characteristics were relatively well balanced between the groups (Table 1, Appendix Tables 1 and 2).

**Table 1:** Patient and treatment characteristics.

Characteristic	Talquetamab (*n* = 28)	Teclistamab (*n* = 37)	IMiD/PI/CD38 combinations (*n* = 43)	Other (*n* = 31)	*P*
**Age, median (range)**	63 (40–78)	64 (40–78)	59 (44–79)	61 (40–78)	0.23
**Female sex,** ***n*** **(%)**	17 (61)	15 (40)	17 (40)	10 (32)	0.15
**CAR T product,** ***n*** **(%)**					0.69
Ide-cel	26 (93)	36 (97)	39 (91)	29 (94)	
Cilta-cel	2 (7)	1 (3)	4 (9)	2 (6)	
**R-ISS,** ***n*** **(%)**					0.57
I	8 (29)	5 (13)	9 (21)	3 (10)	
II	12 (42)	18 (49)	18 (42)	17 (55)	
III	8 (29)	14 (38)	16 (37)	11 (35)	
**Refractory status before CAR T,** ***n*** **(%)**					
Triple-class	23 (82)	33 (89)	36 (84)	23 (74)	0.44
Penta	15 (54)	20 (54)	18 (42)	11 (36)	0.35
**BCMA-directed therapy exposure before CAR T,** ***n*** **(%)**	8 (29)	5 (14)	8 (19)	5 (16)	0.46
**Lines of therapies before CAR T, median (range)**	7 (3–14)	7 (4–14)	6 (4–14)	7 (4–15)	0.81
**MyCARe risk category,** ***n*** **(%)**					0.59
Low	7 (25)	6 (16)	4 (9)	5 (16)	
Intermediate	18 (64)	26 (70)	31 (72)	19 (61)	
High	3 (11)	5 (14)	8 (19)	7 (23)	
**ECOG,** ***n*** **(%)**					0.02
0	7 (25)	7 (19)	4 (9)	4 (13)	
1	19 (68)	25 (68)	28 (65)	27 (87)	
2	2 (7)	5 (13)	11 (26)	0 (0)	
**Time to first relapse after CAR T in months, median (range)**	5.0 (0.8–22.4)	7.3 (1.0–24.6)	3.9 (0.4–17.8)	3.3 (0.8–24.9)	0.02
**Extramedullary relapse,** ***n*** **(%)**	14 (50)	19 (51)	22 (51)	18 (58)	0.92

*IMiD* immunomodulatory drug, *n* number, *R-ISS* revised International Scoring System, *CAR T* chimeric antigen receptor T-cell therapy, *MyCARe* Myeloma CAR T Relapse model (as previously described [6]), *BCMA* B-cell maturation antigen.

Median time to first relapse/progression from CAR T-cell infusion was significantly different between the 4 salvage groups, with 5 months for the talquetamab group, 7.3 months for the teclistamab group, 3.9 months for the IMiDs/PIs/CD38 group, and 3.3 months for others (*P* = 0.02).

Out of the total cohort of 139 patients, 56 (40%) received another subsequent line of salvage therapy of whom 8 (14%) received talquetamab, 11 (20%) received teclistamab, 12 (21%) received chemotherapy, 15 (27%) received IMiDs/PIs/CD38 combinations. Four patients received talquetamab after teclistamab, while 1 patient received teclistamab after talquetamab (Appendix Table 3).

### Salvage therapy with bispecific antibodies induces deep responses and improves outcome

**Fig. 1:**
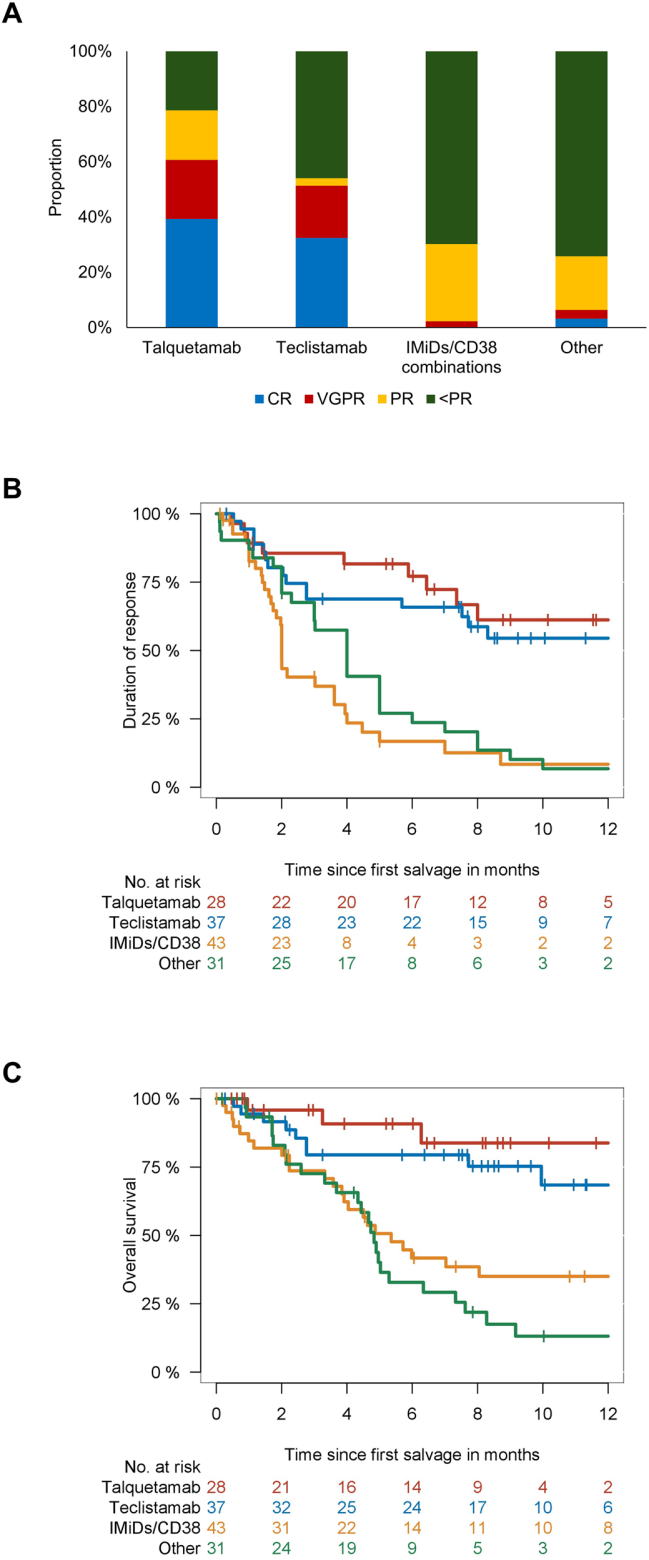
Response and outcome after relapse from CAR T treatment. Response (**A**), duration of response (**B**), and overall survival (**C**) according to first line salvage regimen. Abbreviations: CR complete response; VGPR very good partial response; PR partial response.

Response to first salvage treatment was significantly different, with talquetamab and teclistamab showing better responses (*P* < 0.001; Fig. 1A). Overall response rate was 79% for talquetamab, 64% for teclistamab, 30% for IMiDs/PIs/CD38, and 26% for the rest. Complete response was seen in 39% of the talquetamab group, 32% of the teclistamab group, 0% of the IMiDs/PIs/CD38 group, and 3% of the rest. Very good partial responses were highest for talquetamab (21%), followed by teclistamab (19%). Duration of response as well as median OS was significantly improved with bispecific antibodies (*P* < 0.001, respectively; Fig. 1B, C).

Median OS was not reached for the talquetamab and for the teclistamab group versus 5.4 months (95% CI, 3.7–7.0 months) for IMiDs/PIs/CD38 combinations versus 4.8 months (95% CI, 4.3–5.4 months) for the others.

Importantly, a deeper response to the initial salvage therapy was strongly correlated with extended overall survival (OS) (*P* < 0.001), and this relationship remained consistent irrespective of the applied treatment regimen (*P* < 0.001). All patients with CR to first salvage were alive at last follow-up, and only 2 out of 15 patients with VGPR died at last follow-up (median OS not reached), while median OS for PR or less than PR was 7 months and 5 months, respectively (Appendix Fig. 4A). In particular, similar outcome was observed for bispecific antibodies and other strategies who showed less than PR after first salvage (*P* = 0.51, Appendix Fig. 5A).

Corresponding to better responses and outcome, fewer patients with bispecific antibodies received subsequent lines of treatment (*P* = 0.002), with 21% of the talquetamab group, 39% of the teclistamab, 56% of the IMiDs/PIs/CD38 combinations group, and 68% of the others receiving subsequent treatment after first salvage.

### Bispecific antibodies may overcome the poor prognosis extramedullary relapse

**Fig. 2:**
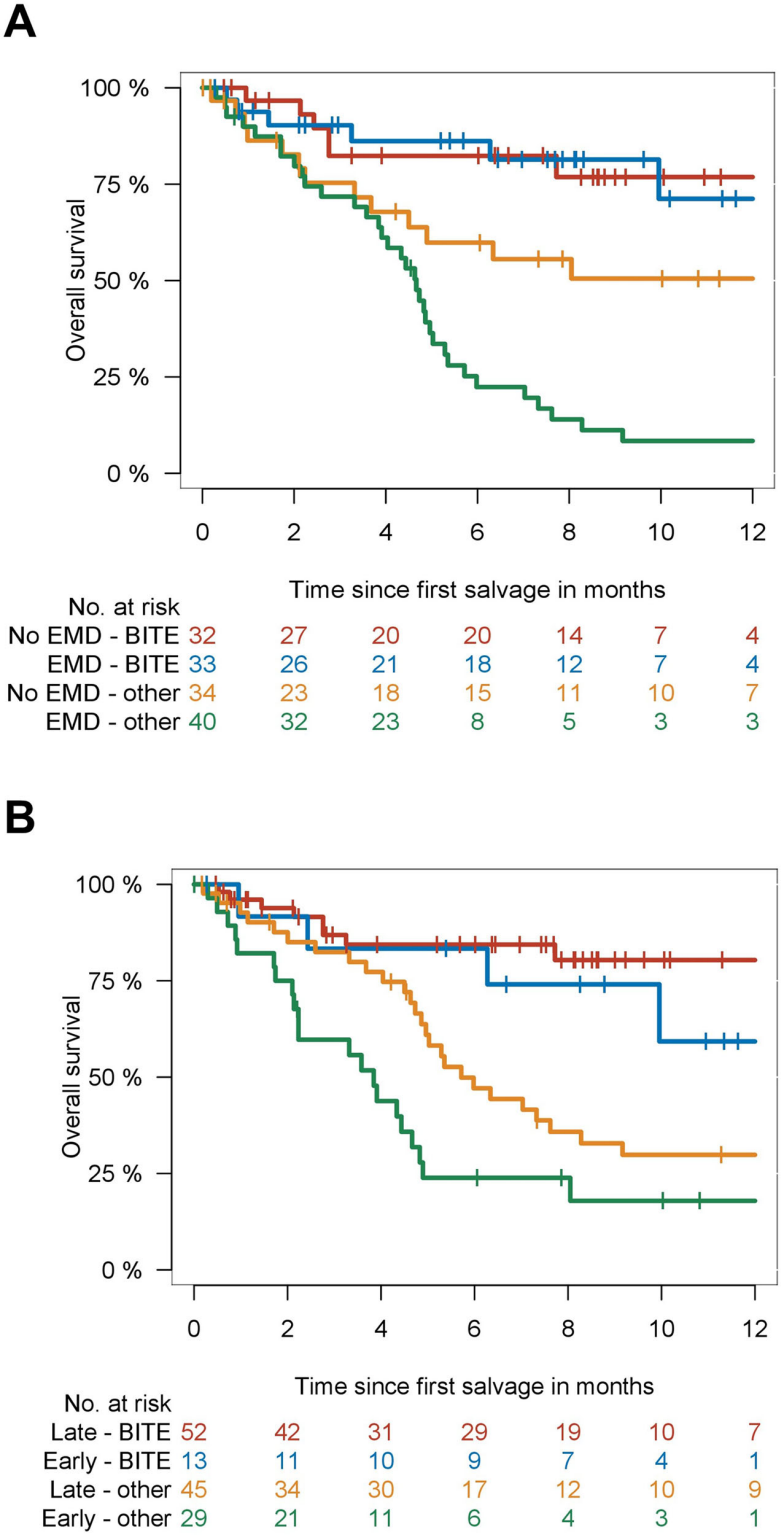
Outcome after CAR T treatment in defined risk categories. Overall survival after first-line salvage according to type of relapse (**A**) and time of relapse (**B**) for comparison of bispecific antibodies and other salvage therapies. Type of relapse was categorized as presence or absence of extramedullary disease (EMD). Time of relapse was categorized as early (within 3 months after CAR T-cell infusion) and late (after 3 months).

Talquetamab and teclistamab seemed to overcome the poor prognosis of EMD relapse, with similar outcome for both EMD versus non-EMD relapse (Fig. 2A). In contrast, EMD relapse was associated with significantly shorter OS versus non-EMD relapse for IMiDs/PIs/CD38 (4.6 months versus not reached; *P* = 0.03) and other treatments (4.7 months versus 6.3 months *P* = 0.06).

### Bispecific antibodies are effective in early and late post-CAR T relapse

With regards to time of relapse after CAR T-cell infusion, bispecific antibodies were associated with better OS for earlier relapse within 5 months after CAR T-cell infusion (*P* = 0.01) as well as for late relapse (*P* = 0.02). The median OS for early relapse was not reached for talquetamab, 10 months for teclistamab, 3.8 months (95% CI, 1.6–6.1 months) for IMiDs/PIs/CD38 combinations, and 4.7 months (95% CI, 4.1–5.2 months) for others. For late relapse, median OS was not reached for both talquetamab and teclistamab versus 7.0 months (95% CI, 0–19.3 months) for IMiDs/PIs/CD38 combinations versus 5.0 months (95% CI, 2.9–7.2 months) for others (Fig. 2B).

### Bispecific antibodies improve outcome across subgroups

**Fig. 3:**
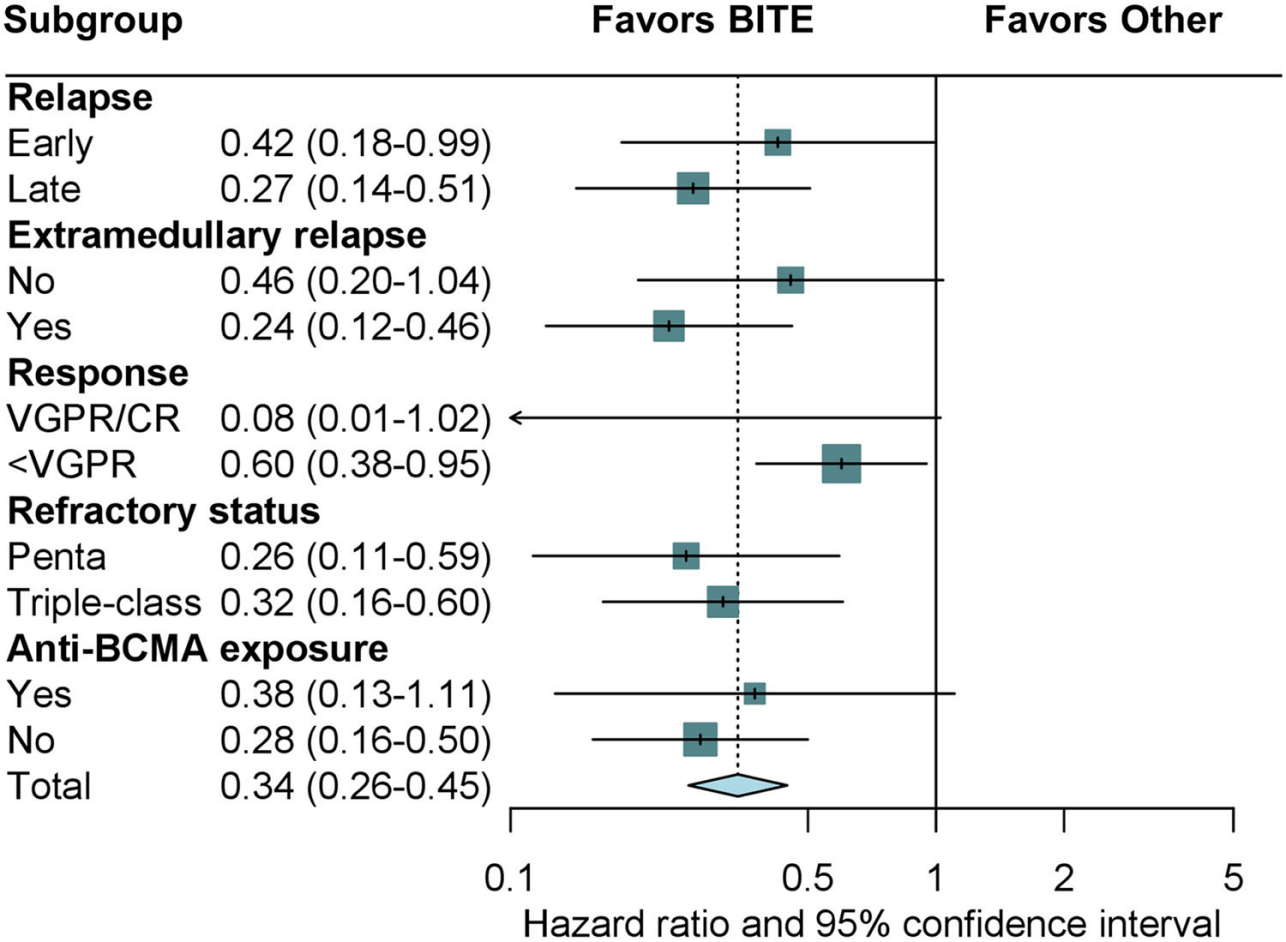
Subgroup analysis on risk of death for the comparison of bispecific antibodies and other salvage therapies. Abbreviations: BITE bispecific antibody, CR complete response, VGPR very good partial response, BCMA B-cell maturation antigen.

Finally, subgroup analysis with cause-specific hazards showed a reduced risk for death for bispecific antibodies across all relevant subgroups (Fig. 3), including EMD relapse, different refractory status, and time of relapse. Bispecific antibodies were associated with reduced risk of death by 76% in patients with EMD and by 58% in patients with early relapse within 3 months after CAR T-cell infusion.

Multivariable modelling identified both talquetamab and teclistamab as independent predictors of improved OS (Table 2). Last, we designed multiple multivariable models, using time to relapse at different cutoff or as continuous variable, confirming the independently improved OS with the use of talquetamab or teclistamab (Appendix Table 4).

**Table 2:** Multivariable model on post-relapse survival.

Factor	Hazard ratio	95% confidence interval	*P*
**Model 1** Concordance = 0.74			
**Treatment group**			
Talquetamab	Reference		
Teclistamab	2.43	0.66–9.05	0.18
IMiDs/PIs/CD38	6.05	1.79–20.39	0.004
Other	9.34	2.76–31.59	<0.001
**Relapse type**			
No EMD	Reference		
EMD	2.53	1.45–4.43	0.001
**Time of relapse**			
Early, <3 months	Reference		
Late, >3 months	0.46	0.27–0.80	0.006
**Penta-refractoriness**	0.89	0.52–1.50	0.65
**ECOG**			
0	Reference		0.73
1	1.46	0.52–2.51	0.05
2	2.76	1.02–7.05	
**Response to 1**^**st**^ **salvage**			
CR/VGPR	Reference		
PR or less	2.54	1.45–3.73	0.001

*IMiD* immunomodulatory drug, *n* number, *EMD* extramedullary disease, *CR* complete response, *VGPR* very good partial response, *PR* partial response.

## Discussion

This is the first international multicenter study in RRMM patients with relapse after BCMA-directed CAR T-cell therapies incorporating all currently approved immunotherapies, with 2 significant findings. First, the outcome of patients is affected by the timing of relapse as well as type of relapse, with early relapse within 5 months after CAR T infusion and relapse with EMD were associated with dismal OS. Second, bispecific antibodies were associated with improved responses and OS compared to other strategies, with the potential to overcome poor prognosis of early relapse and EMD.

Since its approval, CAR T-cell therapy is changing the landscape of the treatment algorithm for RRMM, while the reality of CAR T-cell therapy failure came into focus as more patients received the treatment and approximately 50% experienced relapse within first 12 months after CAR T-cell infusion [15, 16]. Moreover, in the pivotal phase 1 trial investigating the BCMA-directed CAR bb2121, even more than half of all patients had disease progression at 1 year after infusion, even in patients who experienced initial response and including patients without measurable residual disease [17]. Currently, there is no established standard of care for salvage therapies after CAR T cell therapy. Past studies mainly analyzed academic CAR T-cell products and excluded the impact of the anti-GPCR5D bispecific antibody talquetamab [18, 19]. One study reported a 43% ORR among 76 patients, with an ORR of 91% for T-cell–engaging therapies [19]. However, data on first salvage therapies were limited by patient numbers and regimen heterogeneity. Our study included for the first time real-world practices with currently approved therapies, showing that first-line bispecific antibodies are associated with deeper responses and improved OS compared to other first salvage regimens.

One key controversy involves the timing of bispecific antibodies post-CAR T-cell therapy, based on two hypotheses: antigen loss and immune cell exhaustion. The first is based on studies that have shown that BCMA expression in tumor cell populations is heterogeneous and that a relapse after BCMA-directed CAR T-cell therapy may be caused by the selection of clones that lack BCMA expression [20]. Further analysis of patients with myeloma who exhibit BCMA antigen loss suggested del16p appeared to be associated with concurrent del17p mutations, suggesting that resistance to BCMA-directed immunotherapies is more likely to develop in TP53-mutated myeloma [21]. Targeting alternative antigens like GPRC5D may overcome BCMA antigenic loss [22]. In line with this hypothesis, we showed that talquetamab induced the deepest responses and prolonged survival. However, a direct comparison with teclistamab was not significantly different and larger studies are needed to evaluate both therapies.

In addition to tumor-intrinsic factors, T-cell exhaustion is a key obstacle to durable responses. Recent data have elucidated the importance of CAR T-cell expansion and cellular dynamics after treatment [23]. Further, the gradual T-cell exhaustion, combined with a lack of persistence may be particularly implicated in patients whose T-cells were collected after several lines of therapies [24–26], due to an immunosuppressive microenvironment characterized by increased numbers of monocytes and suppressed CD8 + T cell as well as natural killer cell function [27]. Therefore, early application of another immunotherapy post-CAR T-cell therapy might be ineffective. Importantly, we showed that talquetamab and teclistamab were the best options and showed excellent post-relapse median OS even in patients with early relapse (within 3 months or 5 months after CAR T-cell infusion). However, the varying time intervals between relapse post-CAR T-cell therapy and the initiation of salvage therapies may have introduced a selection bias, potentially favoring patients with less aggressive disease for subsequent bispecific antibody treatments.

Treatment of EMD remains an unmet clinical need, with previous therapies showing disappointing responses and survival outcomes [28, 29]. While CAR T-cell therapy has shown remarkable responses, EMD is associated with worse outcomes compared to non-EMD at the time of infusion [6, 12, 13]. Data on EMD relapse post-CAR T-cell therapy were unclear until now. Our study showed that EMD at relapse is linked to poor prognosis, while bispecific antibodies might overcome this with similar responses and survival.

We acknowledge limitations, mostly due to the retrospective design of our study. The recent approval of cilta-cel limited our ability to evaluate a different post-CAR T-cell therapy relapse phenotype due to the small number of patients and short follow-up. Therefore, our results may not be applicable for patients relapsing after cilta-cel and future studies are needed to confirm our findings. Furthermore, evaluating BCMA mutations, along with gene and protein expression, should inform treatment decisions and antigen switching, e.g. in patients with biallelic loss of BCMA. Unfortunately, structured assessments of BCMA mutations are limited to a few centers. Thus, future trials exploring sequencing strategies for T-cell engaging therapies should incorporate assessments of BCMA and/or GPRC5D mutations. Additionally, integrating comprehensive adverse event reporting, currently absent in this study, would strengthen safety evaluations.

In conclusion, this is the first study to show that the bispecific antibodies talquetamab and teclistamab induce deep responses and improve survival after relapse following currently approved BCMA-directed CAR T-cell therapy, irrespective of the poor prognostic factors including time of relapse and extramedullary relapse. Bispecific antibodies should be considered a standard-of-care for patients relapsing after CAR T-cell therapy for RRMM.

## Supplementary information

Supplemental material

## Data Availability

Access to anonymized clinical data might be granted upon reasonable request to the corresponding author.

## References

[1] Gagelmann N, Riecken K, Wolschke C, Berger C, Ayuk FA, Fehse B, et al. Development of CAR-T cell therapies for multiple myeloma. Leukemia. 2020;34:2317–32. 10.1038/s41375-020-0930-x. e-pub ahead of print 20200622 32572190 10.1038/s41375-020-0930-x

[2] Lin Y, Qiu L, Usmani S, Joo CW, Costa L, Derman B et al. Consensus guidelines and recommendations for the management and response assessment of chimeric antigen receptor T-cell therapy in clinical practice for relapsed and refractory multiple myeloma: a report from the International Myeloma Working Group Immunotherapy Committee. *Lancet Oncol* 2024. e-pub ahead of print 20240528; 10.1016/S1470-2045(24)00094-9 10.1016/S1470-2045(24)00094-9 38821074

[3] Munshi NC, Anderson LD Jr, Shah N, Madduri D, Berdeja J, Lonial S, et al. Idecabtagene Vicleucel in Relapsed and Refractory Multiple Myeloma. N. Engl J Med. 2021;384:705–16. 10.1056/NEJMoa2024850 33626253 10.1056/NEJMoa2024850

[4] Berdeja JG, Madduri D, Usmani SZ, Jakubowiak A, Agha M, Cohen AD, et al. Ciltacabtagene autoleucel, a B-cell maturation antigen-directed chimeric antigen receptor T-cell therapy in patients with relapsed or refractory multiple myeloma (CARTITUDE-1): a phase 1b/2 open-label study. Lancet. 2021;398:314–24. 10.1016/S0140-6736(21)00933-8. e-pub ahead of print 20210624 34175021 10.1016/S0140-6736(21)00933-8

[5] Hansen DK, Sidana S, Peres LC, Colin Leitzinger C, Shune L, Shrewsbury A, et al. Idecabtagene Vicleucel for Relapsed/Refractory Multiple Myeloma: Real-World Experience From the Myeloma CAR T Consortium. J Clin Oncol. 2023;41:2087–97. 10.1200/JCO.22.01365. e-pub ahead of print 20230109 36623248 10.1200/JCO.22.01365 PMC10082273

[6] Gagelmann N, Dima D, Merz M, Hashmi H, Ahmed N, Tovar N, et al. Development and Validation of a Prediction Model of Outcome After B-Cell Maturation Antigen-Directed Chimeric Antigen Receptor T-Cell Therapy in Relapsed/Refractory Multiple Myeloma. *J Clin Oncol* 2024: JCO2302232. e-pub ahead of print 20240215; 10.1200/JCO.23.02232 10.1200/JCO.23.02232 PMC11095856 38358946

[7] Cappell KM, Kochenderfer JN. Long-term outcomes following CAR T cell therapy: what we know so far. Nat Rev Clin Oncol. 2023;20:359–71. 10.1038/s41571-023-00754-1. e-pub ahead of print 20230413 37055515 10.1038/s41571-023-00754-1 PMC10100620

[8] Lee H, Ahn S, Maity R, Leblay N, Ziccheddu B, Truger M, et al. Mechanisms of antigen escape from BCMA- or GPRC5D-targeted immunotherapies in multiple myeloma. Nat Med. 2023;29:2295–306. 10.1038/s41591-023-02491-5. e-pub ahead of print 20230831 37653344 10.1038/s41591-023-02491-5 PMC10504087

[9] Rodriguez-Otero P, Usmani S, Cohen AD, van de Donk N, Leleu X, Gallego Perez-Larraya J, et al. International Myeloma Working Group immunotherapy committee consensus guidelines and recommendations for optimal use of T-cell-engaging bispecific antibodies in multiple myeloma. Lancet Oncol. 2024;25:e205–e216. 10.1016/S1470-2045(24)00043-3 38697166 10.1016/S1470-2045(24)00043-3

[10] Lee DW, Santomasso BD, Locke FL, Ghobadi A, Turtle CJ, Brudno JN, et al. ASTCT Consensus Grading for Cytokine Release Syndrome and Neurologic Toxicity Associated with Immune Effector Cells. Biol Blood Marrow Transpl. 2019;25:625–38. 10.1016/j.bbmt.2018.12.758. e-pub ahead of print 20181225 10.1016/j.bbmt.2018.12.758 PMC12180426 30592986

[11] Kumar S, Paiva B, Anderson KC, Durie B, Landgren O, Moreau P, et al. International Myeloma Working Group consensus criteria for response and minimal residual disease assessment in multiple myeloma. Lancet Oncol. 2016;17:e328–e346. 10.1016/S1470-2045(16)30206-6 27511158 10.1016/S1470-2045(16)30206-6

[12] Dima D, Abdallah AO, Davis JA, Awada H, Goel U, Rashid A, et al. Impact of Extraosseous Extramedullary Disease on Outcomes of Patients with Relapsed-Refractory Multiple Myeloma receiving Standard-of-Care Chimeric Antigen Receptor T-Cell Therapy. Blood Cancer J. 2024;14:90 10.1038/s41408-024-01068-w. e-pub ahead of print 20240531 38821914 10.1038/s41408-024-01068-w PMC11143360

[13] Zanwar S, Sidana S, Shune L, Puglianini OC, Pasvolsky O, Gonzalez R, et al. Impact of extramedullary multiple myeloma on outcomes with idecabtagene vicleucel. J Hematol Oncol. 2024;17:42 10.1186/s13045-024-01555-4. e-pub ahead of print 20240606 38845015 10.1186/s13045-024-01555-4 PMC11157748

[14] Gagelmann N, Ayuk FA, Klyuchnikov E, Wolschke C, Berger SC, Kroger N. Impact of high-risk disease on the efficacy of chimeric antigen receptor T-cell therapy for multiple myeloma: a meta-analysis of 723 patients. Haematologica. 2023;108:2799–802. 10.3324/haematol.2022.282510. e-pub ahead of print 20231001 36815380 10.3324/haematol.2022.282510 PMC10542827

[15] Gagelmann N, Ayuk F, Atanackovic D, Kroger N. B cell maturation antigen-specific chimeric antigen receptor T cells for relapsed or refractory multiple myeloma: A meta-analysis. Eur J Haematol. 2020;104:318–27. 10.1111/ejh.13380. e-pub ahead of print 20200120 31883150 10.1111/ejh.13380

[16] St Martin Y, Franz JK, Agha ME, Lazarus HM. Failure of CAR-T cell therapy in relapsed and refractory large cell lymphoma and multiple myeloma: An urgent unmet need. Blood Rev. 2023;60:101095 10.1016/j.blre.2023.101095. e-pub ahead of print 20230429 37173224 10.1016/j.blre.2023.101095

[17] Raje N, Hege K, Kochenderfer JN. Anti-BCMA CAR T-Cell Therapy in Multiple Myeloma. Reply. N. Engl J Med. 2019;381:e6 10.1056/NEJMc1907520 31314982 10.1056/NEJMc1907520

[18] Reyes KR, Liu YC, Huang CY, Banerjee R, Martin T, Wong SW, et al. Salvage therapies including retreatment with BCMA-directed approaches after BCMA CAR-T relapses for multiple myeloma. Blood Adv. 2024;8:2207–16. 10.1182/bloodadvances.2023012066 38429087 10.1182/bloodadvances.2023012066 PMC11061209

[19] Van Oekelen O, Nath K, Mouhieddine TH, Farzana T, Aleman A, Melnekoff DT, et al. Interventions and outcomes of patients with multiple myeloma receiving salvage therapy after BCMA-directed CAR T therapy. Blood. 2023;141:756–65. 10.1182/blood.2022017848 36327160 10.1182/blood.2022017848 PMC10082354

[20] Samur MK, Fulciniti M, Aktas Samur A, Bazarbachi AH, Tai YT, Prabhala R, et al. Biallelic loss of BCMA as a resistance mechanism to CAR T cell therapy in a patient with multiple myeloma. Nat Commun. 2021;12:868 10.1038/s41467-021-21177-5. e-pub ahead of print 20210208 33558511 10.1038/s41467-021-21177-5 PMC7870932

[21] Da Via MC, Dietrich O, Truger M, Arampatzi P, Duell J, Heidemeier A, et al. Homozygous BCMA gene deletion in response to anti-BCMA CAR T cells in a patient with multiple myeloma. Nat Med. 2021;27:616–9. 10.1038/s41591-021-01245-5. e-pub ahead of print 20210222 33619368 10.1038/s41591-021-01245-5

[22] Smith EL, Harrington K, Staehr M, Masakayan R, Jones J, Long TJ et al. GPRC5D is a target for the immunotherapy of multiple myeloma with rationally designed CAR T cells. *Sci Transl Med* 2019; 11. 10.1126/scitranslmed.aau7746 10.1126/scitranslmed.aau7746 PMC7508042 30918115

[23] Fischer L, Grieb N, Born P, Weiss R, Seiffert S, Boldt A, et al. Cellular dynamics following CAR T cell therapy are associated with response and toxicity in relapsed/refractory myeloma. Leukemia. 2024;38:372–82. 10.1038/s41375-023-02129-y. e-pub ahead of print 20240106 38184754 10.1038/s41375-023-02129-y PMC10844085

[24] Holstein SA, Grant SJ, Wildes TM. Chimeric Antigen Receptor T-Cell and Bispecific Antibody Therapy in Multiple Myeloma: Moving Into the Future. J Clin Oncol. 2023;41:4416–29. 10.1200/JCO.23.00512. e-pub ahead of print 20230720 37471687 10.1200/JCO.23.00512 PMC10522112

[25] Parikh RH, Lonial S. Chimeric antigen receptor T-cell therapy in multiple myeloma: A comprehensive review of current data and implications for clinical practice. CA Cancer J Clin. 2023;73:275–85. 10.3322/caac.21771. e-pub ahead of print 20230110 36627265 10.3322/caac.21771

[26] Long AH, Haso WM, Shern JF, Wanhainen KM, Murgai M, Ingaramo M, et al. 4-1BB costimulation ameliorates T cell exhaustion induced by tonic signaling of chimeric antigen receptors. Nat Med. 2015;21:581–90. 10.1038/nm.3838. e-pub ahead of print 20150504 25939063 10.1038/nm.3838 PMC4458184

[27] Rade M, Grieb N, Weiss R, Sia J, Fischer L, Born P, et al. Single-cell multiomic dissection of response and resistance to chimeric antigen receptor T cells against BCMA in relapsed multiple myeloma. Nat Cancer. 2024. 10.1038/s43018-024-00763-8. e-pub ahead of print 20240419 38641734 10.1038/s43018-024-00763-8

[28] Bhutani M, Foureau DM, Atrash S, Voorhees PM, Usmani SZ. Extramedullary multiple myeloma. Leukemia. 2020;34:1–20. 10.1038/s41375-019-0660-0. e-pub ahead of print 20191127 31776467 10.1038/s41375-019-0660-0

[29] Blade J, Beksac M, Caers J, Jurczyszyn A, von Lilienfeld-Toal M, Moreau P, et al. Extramedullary disease in multiple myeloma: a systematic literature review. Blood Cancer J. 2022;12:45 10.1038/s41408-022-00643-3. e-pub ahead of print 20220321 35314675 10.1038/s41408-022-00643-3 PMC8938478

