# Supplementary material for: Bispecific antibodies targeting BCMA or GPRC5D are highly effective in relapsed myeloma after CAR T-cell therapy
Source: Blood Cancer J. 2024 Dec 5;14(1):214. doi: 10.1038/s41408-024-01197-2 (PMC11618392; doi:10.1038/s41408-024-01197-2)
Supplement: Supplementary file 1 — Supplemental material [file 41408_2024_1197_MOESM1_ESM.docx]

**Supplement for:**

**Bispecific antibodies targeting BCMA or GPRC5D are highly effective in relapsed myeloma after CAR T-cell therapy**

**Content**

Patients and Methods

Limitations

Table 1A. Patient characteristics.

Table 2A. First-line salvage regimens for the four treatment groups.

Table 3A. Second-line salvage regimens for the four treatment groups.

Table 4A. Multivariable models on overall survival.

Figure 1A. Overall survival of the total cohort (A), according to time of relapse (B), presence of extramedullary relapse (C), and MyCARe risk category (D).

Figure 2A. Outcomes of type of relapse in talquetamab first-line salvage.

Figure 3A. Outcomes of type of relapse in teclistamab first-line salvage.

Figure 4A. Overall survival in complete response or VGPR after bispecific antibodies versus other salvage therapies.

Figure 5A. Overall survival in partial response or less after bispecific antibodies versus other salvage therapies.

**Patients and Methods**

This multicenter retrospective observational study included patients infused with commercially available BCMA-directed CAR T-cell therapy for RRMM at 12 international centers.^1^ Conducted in accordance with the Declaration of Helsinki, the study included patients relapsed after CAR T-cell therapy with detailed information on salvage regimens. Treatment response and relapse was graded per current guidelines.^2^ Cytokine release syndrome and neurotoxicity were classified in accordance with existing consensus.^3^

Categorical variables were compared between the four first-line salvage groups using chi-squared test, and continuous variables were compared using the Mann-Whitney test for independent samples. Kaplan-Meier estimates analyzed overall survival (OS), with time-to-event calculations starting from first salvage administration. Duration of response (DOR) was defined as the time of response after salvage therapy until progression or relapse, next salvage, death, or last follow-up. Cox regression was used to calculate cause-specific hazard. We developed a multivariable model within a Cox regression framework, calculating hazard ratios with 95% confidence intervals. Concordance index was used to show utility of the model for prediction of post-salvage OS.^4^

**Limitations**

We acknowledge several limitations, mostly due to the retrospective design of our study. The recent approval of cilta-cel limited our ability to evaluate a different post-CAR T-cell therapy relapse phenotype due to the small number of patients and short follow-up.^5^ Furthermore, choice of subsequent therapy, in absence of systematic evidence so far, may have been influenced by relapse timing, clinical phenotype, cytopenias, physician preference, and access.

**Table 1A. Patient and treatment characteristics.**

| **Characteristic** | **Talquetamab**  **(n=28)** | **Teclistamab (n=37)** | **IMiD/CD38 combinations (n=43)** | **Other**  **(n=31)** | **P** |
| --- | --- | --- | --- | --- | --- |
| **Age, median (range)** | 63 (40-78) | 64 (40-78) | 59 (44-79) | 61 (40-78) | 0.23 |
| **Female sex** | 17 (61) | 15 (40) | 17 (40) | 10 (32) | 0.15 |
| **CAR T product** |  |  |  |  | 0.69 |
| Ide-cel | 26 (93) | 36 (97) | 39 (91) | 29 (94) |  |
| Cilta-cel | 2 (7) | 1 (3) | 4 (9) | 2 (6) |  |
| **R-ISS** |  |  |  |  | 0.57 |
| I | 8 (29) | 5 (13) | 9 (21) | 3 (10) |  |
| II | 12 (42) | 18 (49) | 18 (42) | 17 (55) |  |
| III | 8 (29) | 14 (38) | 16 (37) | 11 (35) |  |
| **Refractory status before CAR T** |  |  |  |  |  |
| Triple-class | 23 (82) | 33 (89) | 36 (84) | 23 (74) | 0.44 |
| Penta | 15 (54) | 20 (54) | 18 (42) | 11 (36) | 0.35 |
| **BCMA-directed therapy exposure before CAR T** | 8 (29) | 5 (14) | 8 (19) | 5 (16) | 0.46 |
| **Lines of therapies before CAR T** | 7 (3-14) | 7 (4-14) | 6 (4-14) | 7 (4-15) | 0.81 |
| **MyCARe risk category** |  |  |  |  | 0.59 |
| Low | 7 (25) | 6 (16) | 4 (9) | 5 (16) |  |
| Intermediate | 18 (64) | 26 (70) | 31 (72) | 19 (61) |  |
| High | 3 (11) | 5 (14) | 8 (19) | 7 (23) |  |
| **ECOG** |  |  |  |  | 0.02 |
| 0 | 7 (25) | 7 (19) | 4 (9) | 4 (13) |  |
| 1 | 19 (68) | 25 (68) | 28 (65) | 27 (87) |  |
| 2 | 2 (7) | 5 (13) | 11 (26) | 0 (0) |  |
| **CRS** |  |  |  |  | 0.75 |
| 0 | 4 (14) | 4 (11) | 8 (19) | 6 (19) |  |
| 1 | 19 (68) | 27 (73) | 31 (72) | 23 (74) |  |
| 2 | 5 (18) | 5 (14) | 4 (9) | 2 (7) |  |
| 3 | 0 (0) | 1 (3) | 0 (0) | 0 (0) |  |
| **ICANS** |  |  |  |  | 0.64 |
| 0 | 27 (96) | 32 (87) | 37 (86) | 28 (90) |  |
| 1 | 1 (4) | 3 (8) | 3 (7) | 2 (7) |  |
| 2 | 0 (0) | 2 (5) | 1 (2) | 1 (3) |  |
| 3 | 0 (0) | 0 (0) | 0 (0) | 0 (0) |  |
| 4 | 0 (0) | 0 (0) | 2 (5) | 0 (0) |  |
| **Time to first relapse after CAR T in months, median (range)** | 5.0 (0.8-22.4) | 7.3 (1.0-24.6) | 3.9 (0.4-17.8) | 3.3 (0.8-24.9) | 0.02 |
| **Extramedullary relapse** | 14 (50) | 19 (51) | 22 (51) | 18 (58) | 0.92 |

**Table 2A. First-line salvage regimens for the four treatment groups.**

| **Salvage category, first line** | **Regimen** |
| --- | --- |
| Talquetamab, n=28 | Talquetamab |
| Teclistamab, n=37 | Teclistamab |
| IMiDs or CD38 or PI combinations, n=43 |  |
| 1 | Sel-Pom-dex |
| 2 | Sel-Carf-dex |
| 3 | Dara-Ven-dex |
| 4 | Sel-Pom-dex |
| 5 | Sel-Carf-dex |
| 6 | Dara-Carf-dex |
| 7 | Cyclo-Pom-dex |
| 8 | Elo-Pom-dex |
| 9 | Dara-Carf-Pom-dex |
| 10 | Dara-Pom-dex |
| 11 | Dara-Ven-dex |
| 12 | Carf-Pom-dex |
| 13 | Doxil-Bor |
| 14 | Dara-Pom+Radiation |
| 15 | Isa-Carf-dex |
| 16 | Dara-Carf-dex |
| 17 | Venetoclax-Carf-dex |
| 18 | Bor-Pom |
| 19 | Venetoclax-Carf-dex |
| 20 | Elo-Pom-dex |
| 21 | Seli-Pom-dex |
| 22 | Elo-Pom-Cyclo-dex |
| 23 | Pom-Cyclo-dex |
| 24 | Seli-Carf-dex |
| 25 | Seli-Pom-dex |
| 26 | Seli-Pom-dex |
| 27 | Isa-Carf-dex |
| 28 | Isa-Carf-dex |
| 29 | Isa-Carf-dex |
| 30 | Dara-KDT-PACE |
| 31 | Cyclo-Bor-Pom-dex |
| 32 | Pom-Bor-dex |
| 33 | Pom-Bor-dex |
| 34 | Seli-Pom-dex |
| 35 | Dara-Ven-dex |
| 36 | Isa-Carf-dex |
| 37 | Lenalidomid maintenance |
| 38 | Seli-Bor-dex |
| 39 | Isa-Carf |
| 40 | Bor-Pom-dex |
| 41 | Seli-Pom-dex |
| 42 | Seli-Pom-dex |
| 43 | Pom-dex |
| Other, n=31 |  |
| 1 | Radiation |
| 2 | Radiation |
| 3 | DCEP |
| 4 | VTD-PACE |
| 5 | Auto SCT |
| 6 | DCEP |
| 7 | Allogeneic BCMA CAR-T |
| 8 | PACE |
| 9 | KD-PACE |
| 10 | DCEP |
| 11 | KD-PACE |
| 12 | DV-PACE |
| 13 | DCEP |
| 14 | Venetoclax |
| 15 | Radiation |
| 16 | Hospice, no further treatmens |
| 17 | Hospice, no further treatmens |
| 18 | DCEP |
| 19 | DCEP |
| 20 | Allo SCT |
| 21 | IT Methotrexate plus IT Cytarabine, VD-PACE |
| 22 | VD-PACE |
| 23 | Elranatamab |
| 24 | Blenrep |
| 25 | Blenrep |
| 26 | Radiation |
| 27 | Melflufen |
| 28 | Radiation |
| 29 | Bendamustin/Prednisolon |
| 30 | Decitabine + Venetoclax |
| 31 | Auto SCT |

**Table 3A. Second-line salvage regimens for the four treatment groups.**

| **Salvage category, first line** | **Regimen, second line** |
| --- | --- |
| Talquetamab | Dara-KdT-PACE |
|  | Radiotherapy |
|  | Seli-Bor-dex |
|  | Teclistamab |
| Teclistamab | Cyclo-Car-dex |
|  | DCEP |
|  | DCEP |
|  | Elo-Pom-dex |
|  | FVD |
|  | Radiotherapy |
|  | Seli-Bor-dex |
|  | Talquetamab |
|  | Talquetamab |
|  | Talquetamab |
|  | Talquetamab |
|  | VD-PACE |
|  | Ven-Cyclo-dex |
| IMiDs or CD38 or PI combinations | Benda-dex |
|  | Seli-Bor |
|  | Cyclo-dex |
|  | Dara-Cyclo-Bor-dex |
|  | DCEP |
|  | Elo-Ven-dex |
|  | Best supportive care |
|  | Isa-Pom-dex |
|  | Seli-Car-dex |
|  | Seli-Car-dex |
|  | Seli-Pom-dex |
|  | Talquetamab |
|  | Talquetamab |
|  | Talquetamab |
|  | Talquetamab |
|  | Teclistamab |
|  | Teclistamab |
|  | Teclistamab |
|  | Teclistamab |
|  | Teclistamab |
|  | Thal-dex |
|  | VDT-PACE |
|  | VDT-PACE |
| Other | Cyclo-Pom-dex |
|  | Cyclo-Pom-dex |
|  | D-PACE |
|  | DV-PACE |
|  | Elo-Bor-Pom-dex |
|  | Pom-Cyclo-dex |
|  | Seli-Car-dex |
|  | Talquetamab |
|  | Teclistamab |
|  | Teclistamab |
|  | Teclistamab |
|  | Teclistamab |
|  | Teclistamab |
|  | VD-PACE |

**Table 4A. Multivariable models on overall survival.**

| **Factor** | **Hazard ratio** | **95% confidence interval** | **P** |
| --- | --- | --- | --- |
| **Model 1**  Concordance=0.74 |  |  |  |
| **Treatment group** |  |  |  |
| Talquetamab | Reference |  |  |
| Teclistamab | 2.43 | 0.66-9.05 | 0.18 |
| IMiDs/CD38/PI | 6.05 | 1.79-20.39 | 0.004 |
| Other | 9.34 | 2.76-31.59 | <0.001 |
| **Relapse type** |  |  |  |
| No EMD | Reference |  |  |
| EMD | 2.53 | 1.45-4.43 | 0.001 |
| **Time of relapse** |  |  |  |
| Early, <3 months | Reference |  |  |
| Late, >3 months | 0.46 | 0.27-0.80 | 0.006 |
| **Penta-refractoriness** | 0.89 | 0.52-1.50 | 0.65 |
| **ECOG** |  |  |  |
| 0 | Reference |  | 0.73 |
| 1 | 1.46 | 0.52-2.51 | 0.05 |
| 2 | 2.76 | 1.02-7.05 |  |
| Response to 1^st^ salvage |  |  |  |
| CR/VGPR | Reference |  |  |
| PR or less | 2.54 | 1.45-3.73 | 0.001 |
| **Model 2**  Concordance=0.73 |  |  |  |
| **Treatment group** |  |  |  |
| Talquetamab | Reference |  |  |
| Teclistamab | 2.18 | 0.58-8.23 | 0.25 |
| IMiDs/CD38/PI | 4.63 | 1.37-15.67 | 0.01 |
| Other | 7.07 | 2.09-23.86 | 0.001 |
| **Relapse type** |  |  |  |
| No EMD | Reference |  |  |
| EMD | 2.29 | 1.31-4.01 | 0.004 |
| **Time of relapse, continuous** | 0.95 | 0.89-1.01 | 0.12 |
| **Penta-refractoriness** | 0.77 | 0.45-1.31 | 0.33 |
| **ECOG** |  |  |  |
| 0 | Reference |  |  |
| 1 | 1.26 | 0.55-2.89 | 0.59 |
| 2 | 2.94 | 1.04 | 0.04 |
| Response to 1^st^ salvage |  |  |  |
| CR/VGPR | Reference |  |  |
| PR or less | 2.12 | 1.30-3.46 | 0.003 |
| **Model 3**  Concordance=0.75 |  |  |  |
| **Treatment group** |  |  |  |
| Talquetamab | Reference |  |  |
| Teclistamab | 2.08 | 0.57-7.70 | 0.27 |
| IMiDs/CD38/PI | 5.39 | 1.61-18.07 | 0.006 |
| Other | 8.04 | 2.40-17.00 | <0.001 |
| **Relapse type** |  |  |  |
| No EMD | Reference |  |  |
| EMD | 2.17 | 1.25-3.74 | 0.006 |
| **Time of relapse** |  |  |  |
| Early, <5 months | Reference |  |  |
| Late, >5 months | 0.58 | 0.34-0.99 | 0.04 |
| **Penta-refractoriness** | 0.79 | 0.47-1.33 | 0.37 |
| **ECOG** |  |  |  |
| 0 | Reference |  |  |
| 1 | 1.22 | 0.56-2.68 | 0.62 |
| Response to 1^st^ salvage |  |  |  |
| CR/VGPR | Reference |  |  |
| PR or less | 2.09 | 1.30-3.35 | 0.002 |

**Figure 1A. Overall survival of the total cohort (A), presence of extramedullary relapse (B), according to time of relapse (C), and MyCARe risk category (D).**


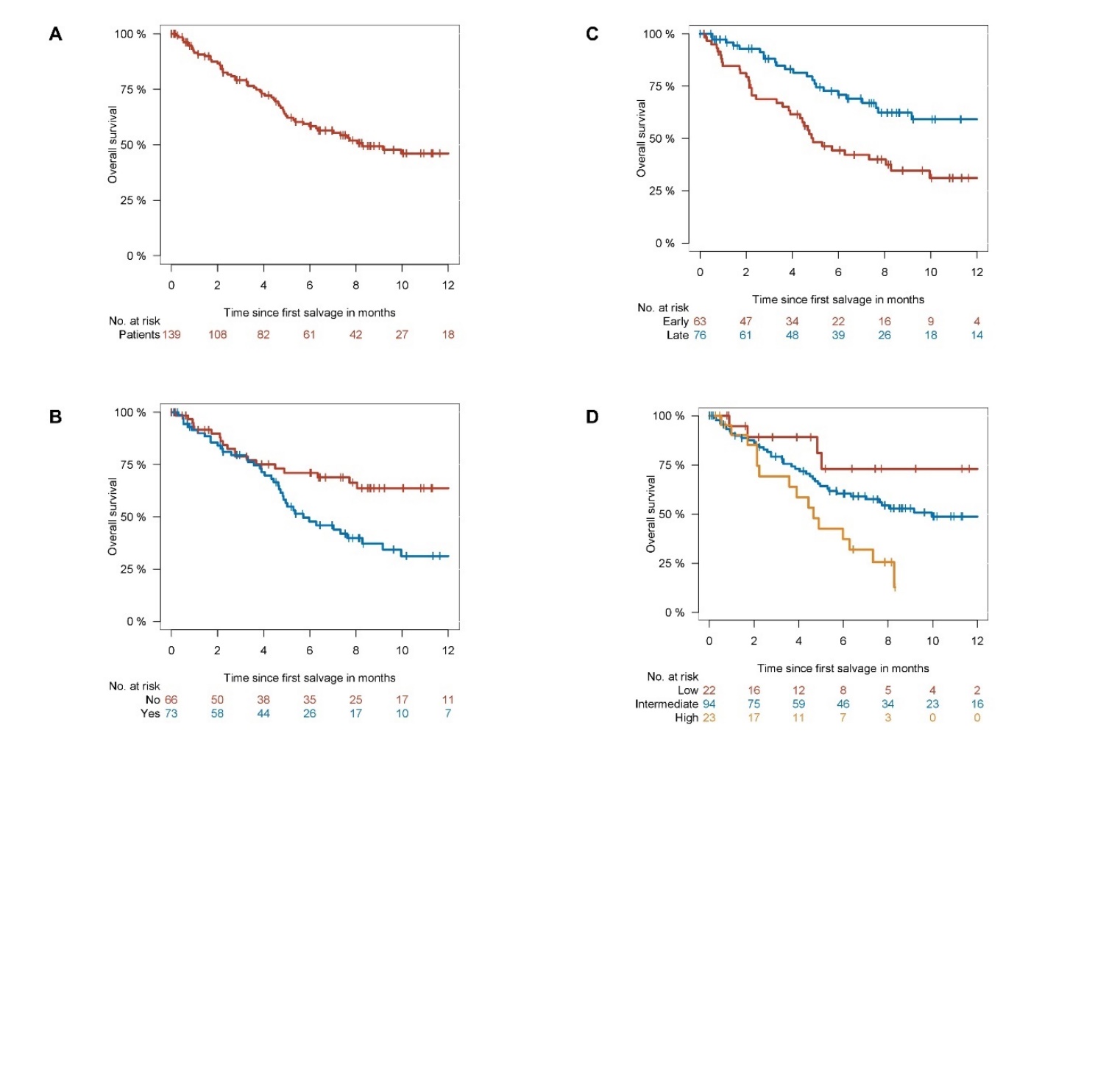


**Figure 2A. Outcomes of type of relapse in talquetamab first-line salvage.**


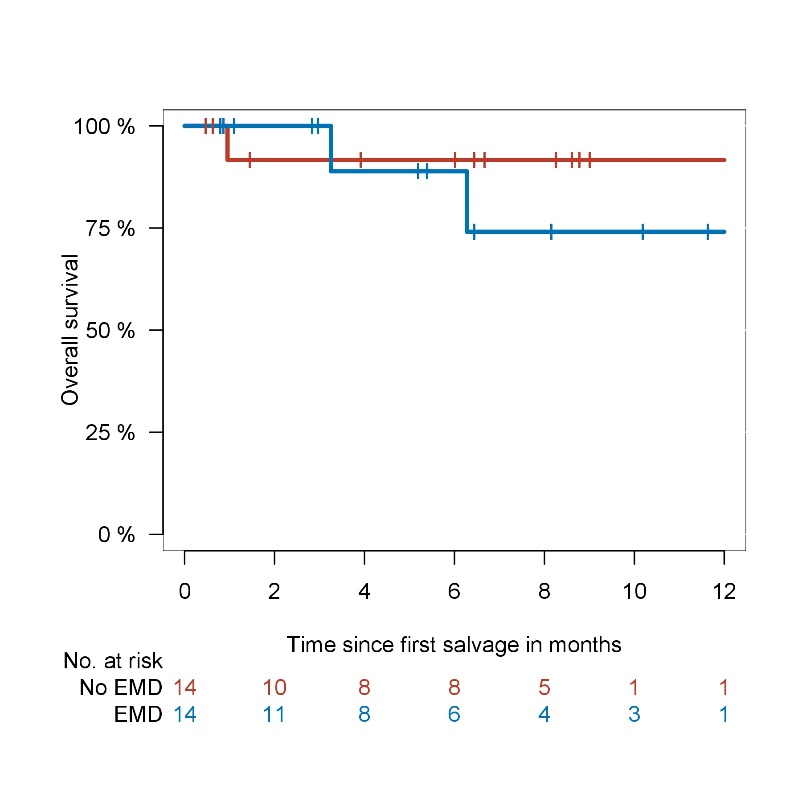


**Figure 3A. Outcomes of type of relapse in teclistmab first-line salvage.**


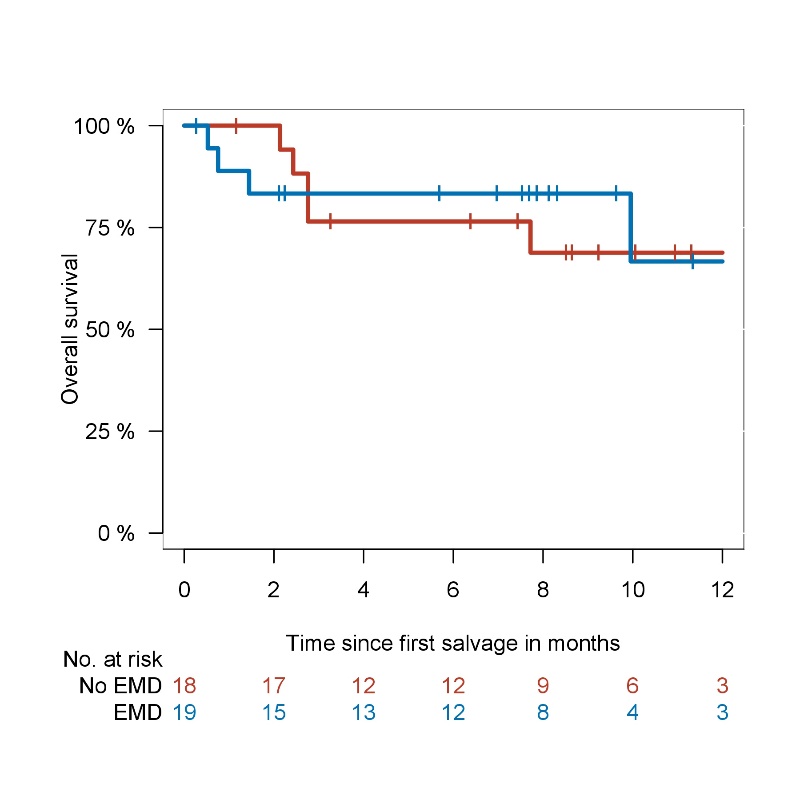


**Figure 4A. Overall survival in complete response or VGPR after bispecific antibodies versus other salvage therapies.**


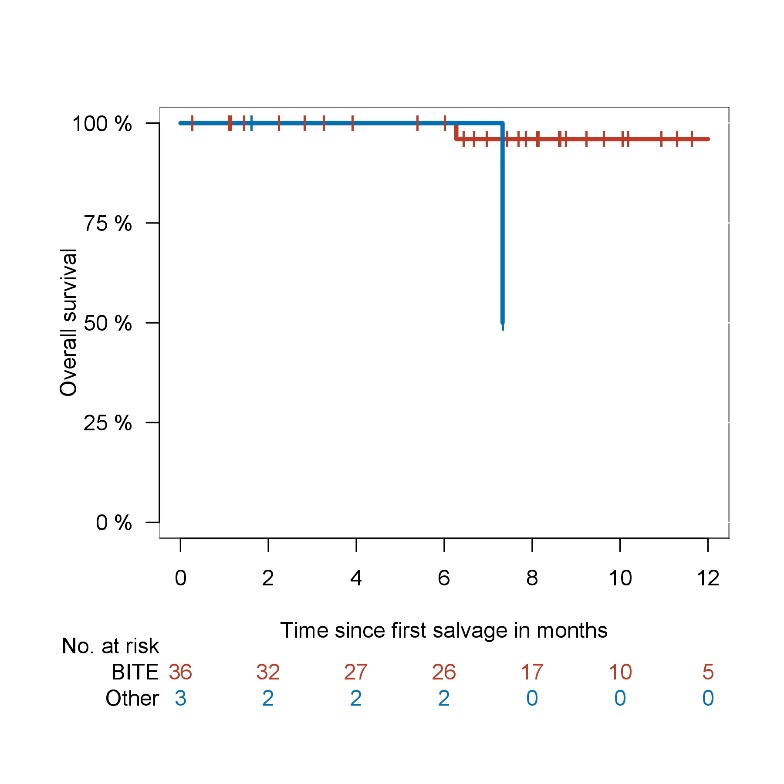


**Figure 5A. Overall survival in partial response or less after bispecific antibodies versus other salvage therapies.**


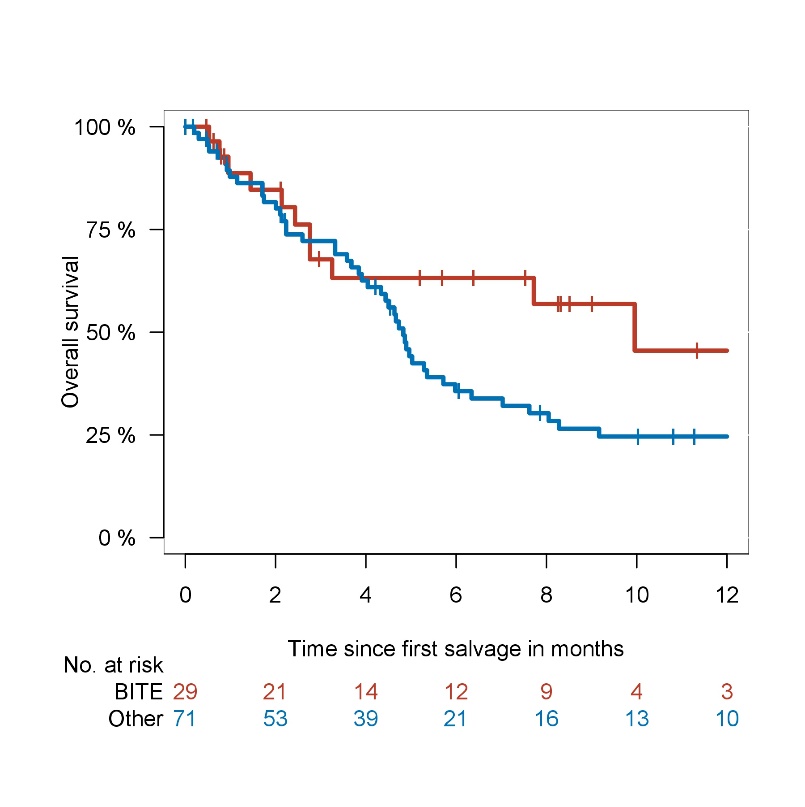


1. Gagelmann N, Dima D, Merz M, et al. Development and Validation of a Prediction Model of Outcome After B-Cell Maturation Antigen-Directed Chimeric Antigen Receptor T-Cell Therapy in Relapsed/Refractory Multiple Myeloma. J Clin Oncol 2024:JCO2302232. DOI: 10.1200/JCO.23.02232.

2. Kumar S, Paiva B, Anderson KC, et al. International Myeloma Working Group consensus criteria for response and minimal residual disease assessment in multiple myeloma. Lancet Oncol 2016;17(8):e328-e346. DOI: 10.1016/S1470-2045(16)30206-6.

3. Lee DW, Santomasso BD, Locke FL, et al. ASTCT Consensus Grading for Cytokine Release Syndrome and Neurologic Toxicity Associated with Immune Effector Cells. Biol Blood Marrow Transplant 2019;25(4):625-638. DOI: 10.1016/j.bbmt.2018.12.758.

4. Harrell FE, Jr., Lee KL, Mark DB. Multivariable prognostic models: issues in developing models, evaluating assumptions and adequacy, and measuring and reducing errors. Stat Med 1996;15(4):361-87. DOI: 10.1002/(SICI)1097-0258(19960229)15:4<361::AID-SIM168>3.0.CO;2-4.

5. Berdeja JG, Madduri D, Usmani SZ, et al. Ciltacabtagene autoleucel, a B-cell maturation antigen-directed chimeric antigen receptor T-cell therapy in patients with relapsed or refractory multiple myeloma (CARTITUDE-1): a phase 1b/2 open-label study. Lancet 2021;398(10297):314-324. DOI: 10.1016/S0140-6736(21)00933-8.
